# Fear and discomfort of children and adolescents during MRI: ethical consideration on research MRIs in children

**DOI:** 10.1038/s41390-020-01277-6

**Published:** 2021-04-20

**Authors:** Regula Everts, Raphaela Muri, Kurt Leibundgut, Valerie Siegwart, Roland Wiest, Maja Steinlin

**Affiliations:** 1 Division of Neuropediatrics, Development and Rehabilitation, Children’s University Hospital, Inselspital, Bern University Hospital, University of Bern, Bern, Switzerland; 2 Department of Diabetes, Endocrinology, Nutritional Medicine and Metabolism, Inselspital, Bern University Hospital, University of Bern, Bern, Switzerland; 3 Department of Pediatric Hematology and Oncology, Children’s University Hospital, Inselspital, Bern University Hospital, University of Bern, Bern, Switzerland; 4 Institute of Diagnostic and Interventional Neuroradiology, Inselspital, Bern University Hospital, University of Bern, Bern, Switzerland

## Introduction

Magnetic resonance imaging (MRI) is the imaging modality of choice for clinical diagnostics and research settings. Despite the fact that unsedated MRI without contrast agents is defined as a minimal-risk procedure,^1–4^ some research review boards consider exposure of healthy children and adolescents to magnetic fields of 1.5 or 3 Tesla only for research purposes ethically unjustifiable. According to the US regulations, minimal risk means that “the probability and magnitude of harm or discomfort anticipated in the research are not greater than those ordinarily encountered in daily life or during the performance of routine physical or psychological examinations or tests”.^3^ Despite that MRI is a low-risk procedure, it might come along with some burden. Determining the level of burden associated with a study protocol is a difficult endeavor, especially in pediatric research.

Only few studies describe fear and discomfort of children during research MRI.^5–9^ The likelihood of discomfort during clinical MRI is reported to be very low with more than 98% children and adolescents stating no or only minimal fear during clinical MRI.^5^ The same study demonstrated that anxiety levels did not differ between MRI and electroencephalography assessments; however, first-ever MRI was related to significantly higher physiological arousal than repeated MRI.^5^ A funtional MRI study compared anxious children to non-anxious children and adults and did not find a difference in their emotional reaction to the MRI.^6^ Children are suggested to experience less discomfort and more enjoyment during MRI than adults.^7^ In older studies, the insertion of a needle for the application of contrast agents, the confined space, loud noise, and the instruction to lay still was suggested to particularly cause anxiety and distress in some children and adolescents.^8,9^ Research projects in developmental neuroscience rarely use contrast agents, particularly not when healthy controls are included. From a psychological point of view, unsedated MRI without contrast agents in pediatrics meets the minimal-risk standard.^4,10^ Various strategies exist which help reduce the level of fear and discomfort during MRI in children (see refs. ^11–13^, and for an overview of such strategies see Table S4). Children showed stronger engagement, more comfort, and more enjoyment than adults^7^ and furthermore rated the MRI as less distressing than their parents did.^9^ These findings highlight the positive side of enrolling children in volunteer-based research MRI.

Likewise, the risk for physical harm in unsedated MRI is very low.^1,4,14^ Radiologist’s associations provide useful information on hazards that need to be considered during MRI and for risk assessment.^15,16^ However, they exclude children and do not separate between clinical and research MRI.^4^ Consequently, international controversies remain whether it is ethically justifiable to perform MRI scans in children and adolescents for research purposes only.

The guidelines of swissethics (Swiss Association of Research Ethics Committees) precise that in research procedures with children “not only the objective risk, but also the subjective experience of the minors plays a role”.^1^ To avoid experience of discomfort and to deal with a supposedly low risk–benefit ratio, some review boards recommend using, instead of healthy controls, MRI exams from “healthy” children having undergone MRI for other reasons (i.e. suspicion of epilepsy, brain tumor, stroke or others). However, these children do not present a healthy sample, receive different MRI sequences than used for research assessments, and are therefore unsuitable as a control group.

To summarize, the review boards’ recommendation to exclude healthy children from MRI studies does not appear feasible and lacks scientific basis. It is rather a worrying obstruction, which hinders new insights into the developing brain.

The aim of the present study is to provide empirical evidence on fear and discomfort during unsedated research MRI without contrast agents in 212 participants aged 7–18 years. We further considered aspects that–based on previous research–are expected to relate to fear and discomfort during MRI such as age,^17^ intelligence,^18^ parental education level,^19^ and cognitive self-control.^20^ Additionally, this study provides data on the intra-individual longitudinal development of fear and discomfort during MRI between childhood and adolescence. Our findings will be helpful for review boards evaluating study protocols including research MRIs in healthy children and adolescents and will enable review boards to base their decision about the risk–benefit ratio on group-level data and hence on solid grounds.

## Methods

Between 2010 and 2018, participants were scanned by doctoral students in psychology who received an extensive introduction and practical training on how to perform MRI in children. Participants became familiar with the noise inside the scanner via audio presentation and were carefully instructed on the MRI procedure in detail before the scan. Participants, who wished for their parents to be close, were scanned with a parent sitting next to them. In between every MR sequence, the examiner talked to the participants over the intercom to make sure that everything is all right.

To capture the extent of children’s fear and discomfort, all participants completed a self-report rating scale immediately after the MRI, such as suggested in a previous study.^21^ Answers were given on a smiley-based scale (0 = no discomfort/no fear, 1 = almost no discomfort/almost no fear, 2 = a little discomfort/a little fear, 3 = considerable discomfort/considerable fear, 4 = high discomfort/high fear), ranging from a friendly laughing smiley (=0) to a very worried smiley (=4). Fear and discomfort were each assessed on a separate scale.

Parental education was chosen as one of the many possible proxies for socio-economic status and was defined as the highest maternal and paternal education level according to the Swiss education system (no graduation = 1, college = 2, college of higher education = 3, university degree = 4). Intelligence was assessed using the WISC-IV (see ref. ^21^; NEMO-Study) and the TONI-4 (see ref. ^22^; Brainfit-Study). Cognitive self-control was captured using an inhibition task (color word interference task^23^). All cognitive assessments and questionnaires were administered on the day of scanning.

In total, 102 healthy controls and 110 patients (7–18 years) were included. All participants were either enrolled in the NEMO-Study^24^ assessing children born very preterm (*n* = 51) and healthy controls (*n* = 49) or the Brainfit-Study,^25^ examining survivors of childhood cancer (*n* = 59) and healthy controls (*n* = 53). For inclusion criteria see the corresponding references.

Both studies took place at the Children’s University Hospital in Bern, Switzerland; study protocols were approved by the local ethics committee. All parents and participants signed written informed consent prior to enrollment if >14 years (for participants <14 years, the legal guardian signed the consent).

Categorical data (MRI questionnaire, parental education) was analyzed using Mann–Whitney *U*-test. *T*-test and Chi-square test was used to analyze group difference concerning age (*T*-test) and sex (Chi-square). To evaluate the relationship between fear, discomfort and sex, parental education, IQ, and cognitive self-control, Spearman correlation was used. For longitudinal analyses, Wilcoxon signed ranks test was applied to compare fear and discomfort in childhood versus adolescence. The level of significance was set to *p* 0.05 (two tailed). Bonferroni correction was applied for correlation analyses setting the *p* value to 0.007.

## Results

Mean age at exam (*t* = 2.409, *p* = 0.017), IQ (*t* = 2.855, *p* = 0.005), and parental education (*U* = 3517.5, *p* = 0.000) differed significantly between patients and controls (Table S1). IQ differed significantly between studies, with preterm-born participants (NEMO-Study, *M* = 100.88) presenting significantly lower IQ than childhood cancer survivors (Brainfit-Study, *M* = 106.14, *t* = 2.674, d.f. = 108, *p* = 0.009). In controls, IQ did not differ between studies (*t* = −0.299, d.f. = 94.097, *p* = 0.766).

**Fig. 1:**
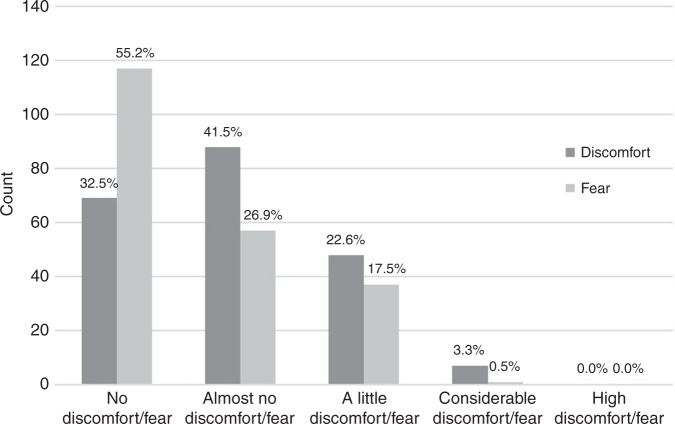
Fear and discomfort in children. Number and percentages of children experiencing fear and discomfort on a scale between 0 (no fear/discomfort) to 4 (high fear/discomfort).

Patients and controls did not differ concerning perceived fear (*U* = 5193.0, *p* = 0.298) and discomfort (*U* = 5432.0, *p* = 0.671, Table S2). The majority of participants experienced no or almost no fear (82.1%) and discomfort (74.0%, Fig. 1). In all, 17.5% experienced a little fear and 22.6% a little discomfort, whereas the rate of considerable fear (0.5%) or discomfort (3.3%) was very low. The maximum score of high fear/discomfort was never given, neither in controls nor in patients. There was a significant sex difference, with discomfort being higher in male than female patients (*U* = 1174.0, *p* = 0.036), whereas the level of fear did not differ between males and females (*U* = 1444.0, *p* = 0.703). In controls, no sex difference occurred in terms of discomfort (*U* = 1244.5, *p* = 0.714) or fear (*U* = 1230.5, *p* = 0.631) during the MRI.

**Fig. 2:**
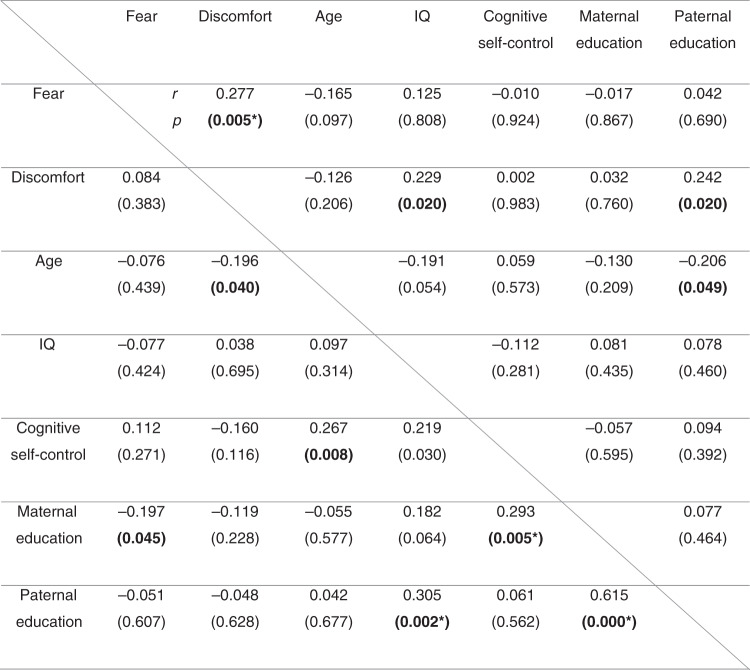
Correlation between output measures (fear and discomfort) and age at exam, IQ, cognitive self-control, and parental education (*n* = 102 controls above the diagonal line; *n* = 110 patients below the diagonal line).

In controls, fear and discomfort was positively associated. In patients, age was negatively associated with discomfort whereas maternal education correlated positively with cognitive self-control and paternal education was positively associated with IQ (Fig. 2).

Longitudinal analysis on the development of fear and discomfort between childhood and adolescence showed that fear during MRI decreased significantly between childhood and adolescence in patients (*z* = −2.36, *p* = 0.018) but not in controls (*z* = −1.22, *p* = 0.222, Table S3). The level of perceived discomfort remained stable between childhood and adolescence in patients (*z* = −0.218, *p* = 0.827) and controls (*z* = −0.922, *p* = 0.356).

## Discussion

We provide empirical data on fear and discomfort associated with unsedated head MRI in 212 children and adolescents aged 7–18 years (without the use of contrast agents). Self-reported fear and discomfort was very low in participants undergoing research MRI with younger patients reporting more fear than older participants. Older participants are likely to have more experience in handling unfamiliar situations and had more opportunities to internalize possible coping strategies than younger participants and might therefore be less prone to a fearful reaction. Based on the present finding, a general exclusion of minors from MRI research studies due to possible discomfort does not appear justified.

Our results show more discomfort in male than in female patients. The literature concludes that the male and female brain differs in structure and function considerably^26^ and that males and females use different ways to solve the same problem and converge on the same behavior,^27^ a fact likely reflected in the sex difference found in our study.

Review boards are often concerned that children do not benefit from participating in neuroimaging research studies and hence presume a low risk–benefit ratio. Our experience and the experience of others^7^ show that MRI is an excellent teaching tool giving children the opportunity to interact with modern medical technology, get an insight into potential career choices, and contribute to research. These are striking benefits that are neglected in the discourse on the risk–benefit ratio of pediatric study participants. By all means, we recommend to formulate elaborated research questions with convincing rationales to clarify the necessity of a study and to justify any minor inconveniences experienced by the children for the sake of scientific advancement.

Recruiting healthy children nowadays is challenging and expensive and often forces researchers to focus merely on the study of ill children. To allow for statements about brain development and reorganization after disease, the comparison with healthy children’s brains is inevitable. For example, the investigation on resting-state functional networks after stroke in childhood needs normative data of healthy controls^28^ or studying characteristics of the working memory network in preterm-born children is impossible without the knowledge on the development of the working memory network in healthy children.^29^ Recently, open access imaging data are available for healthy children and adolescents (i.e. https://data.rocklandsample.rfmh.org). These datasets allow for the inclusion of control data serving as normative information when studying patients. However, technical constraints often hinder the comparison of open access imaging data with imaging sequences from the local site.^30^

Limitations of our study are the following: Children undergoing MRI for clinical reasons might experience more fear and discomfort than participants in our research setting who consented to the study procedure on a voluntary basis and likely exhibit extraordinary motivation to undergo an MRI scan. Additionally, it has been shown that post-MRI anxiety is significantly lower than pre-MRI anxiety,^10,31,32^ pointing towards a positive bias due to relieving feelings after the MRI scan. Whether specific factors such as the noise of the scanner, the restricted space, the presence of a parent or the parenting style contribute to perceived fear and discomfort remains to be investigated in future studies.

To conclude, our data present evidence that the majority of study participants do not suffer from discomfort during the MRI scan. Restraints from research review boards concerning unsedated research MRI without contrast agents in healthy children and adolescents seem unjustified and need to be carefully reconsidered to not impede the advancement of developmental neuroscience.

## Supplementary information

Supplementary material
