# Supplementary material for: Fear and discomfort of children and adolescents during MRI: ethical consideration on research MRIs in children
Source: Pediatr Res. 2021 Apr 20;91(4):720–3. doi: 10.1038/s41390-020-01277-6 (PMC9064788; doi:10.1038/s41390-020-01277-6)
Supplement: Supplementary file 1 — Supplementary material [file 41390_2020_1277_MOESM1_ESM.docx]

| **Supplementary Material**   \| *Table S1*.  **Demographic variables** \| \| \| \| \| \| \|  \| \| --- \| --- \| --- \| --- \| --- \| --- \| --- \| --- \| \|  \| **Patients**  (*n* = 110) \| **Controls**  (*n* = 102) \| U/t/*χ2* \| *z/df* \| *p* \| *d_Cohen_* \| \| \| Study \|  \|  \|  \|  \|  \| \|  \| \| *n* NEMO-Study  *n* Brainfit-Study \| 51  59 \| 49  53 \|  \|  \|  \| \|  \| \| Sex  *n* females (%)  *n* males (%) \| 50 (45.2%)  60 (54.8%) \| 48 (47.1%)  54 (52.9%) \| .055 \| 1 \| .815 \| \|  \| \| Age at exam (years)  *M* (*SD*)  Range \| 10.46 (2.18)  7.22 – 15.85 \| 11.30 (2.85)  7.04 – 18.00 \| 2.433 \| 210 \| .016* \| \| .33 \| \| Parental education † \|  \|  \|  \|  \|  \| \|  \| \| mothers *Md (IQR)* \| 3.00 (1.00) \| 3.00 (1.00) \| 3517.5 \| -3.7 \| .000* \| \|  \| \| fathers *Md (IQR)* \| 3.00 (2.00) \| 4.00 (2.00) \| 3606.0 \| -3.1 \| .002* \| \|  \| \| IQ  *M* (*SD*)  Range \| 103.70 (10.56)  82 - 133 \| 107.91 (10.92)  83 - 132 \| 2.855 \| 210 \| .005* \| \| .39 \| \| Self-control \|  \|  \|  \|  \|  \| \|  \| \| *M* (*SD*)  Range \| 10.11 (2.51)  3-14 \| 10.81 (1.99)  3-14 \| 2.146 \| 185.29 \| .033* \| \| .01 \| \| *Note. M =* Mean; *SD* = Standard Deviation; *Md* = Median; *IQR* = Interquartile Range; *U* = Mann-Whitney *U-*Test; *χ2* = chi-square; *z* = *z*-value; *df* = degrees of freedom; *p* = level of significance, two-tailed; Significant differences are marked with *; † parental education as a proxy for socio-economic status (1 = no graduation, 2 = college, 3 = college of higher education, 4 = university degree); IQ = intelligence quotient, *M* = 100, *SD* = 15. \| \| \| \| \| \| \| \| |
| --- | --- | --- | --- | --- | --- | --- | --- | --- | --- | --- | --- | --- | --- | --- | --- | --- | --- | --- | --- | --- | --- | --- | --- | --- | --- | --- | --- | --- | --- | --- | --- | --- | --- | --- | --- | --- | --- | --- | --- | --- | --- | --- | --- | --- | --- | --- | --- | --- | --- | --- | --- | --- | --- | --- | --- | --- | --- | --- | --- | --- | --- | --- | --- | --- | --- | --- | --- | --- | --- | --- | --- | --- | --- | --- | --- | --- | --- | --- | --- | --- | --- | --- | --- | --- | --- | --- | --- | --- | --- | --- | --- | --- | --- | --- | --- | --- | --- | --- | --- | --- | --- | --- | --- | --- |

| *Table S2*  **Perceived fear and discomfort level during the MRI in patients and controls** | | | | |
| --- | --- | --- | --- | --- |
|  | **Patients**  (*n* = 110) | **Controls**  (*n* = 102) | *U(z)* | *p* |
| Fear, *Md* (*IQR*)  range | 0.00 (1.00)  0 - 2 | 0.00 (1.00)  0 - 3 | 5193.0 (-1.040) | .298 |
| Discomfort, *Md* (*IQR*)  range | 1.00 (1.00)  0 - 3 | 1.00 (2.00)  0 - 3 | 5432.0 (-.425) | .671 |

*Note. Md* = Median; *IQR* = Interquartile Range; *U* = Mann-Whitney U-Test; *z* = z-value; *p* = level of significance, two-tailed.

*Table S3.*

**Longitudinal development of perceived fear and discomfort during MRI**

|  | **Patients** | |  | **Controls** | |  |
| --- | --- | --- | --- | --- | --- | --- |
|  | childhood  (*n* = 34) | adolescence  (*n =* 34) | *z p* | childhood  (*n =* 22) | adolescence  (*n =* 22) | *z p* |
| Fear, *Md* (*IQR*) | 0.00  (2.00) | 0.00  (0.00) | -2.35 .018* | 0.50  (1.00) | 0.00  (1.00) | -1.22 .222 |
| range | 0 - 2 | 0 - 2 |  | 0 - 2 | 0 - 3 |  |
| Discomfort, *Md* (*IQR*) | 1.00  (1.00) | 1.00  (1.00) | -.21 .827 | 1.00  (2.00) | 1.00  (1.00) | -.92 .356 |
| range | 0 - 3 | 0 - 2 |  | 0 - 2 | 0 - 3 |  |

*Note. Md* = Median; *IQR* = Interquartile Range; *U* = Mann-Whitney U-Test; *z* = z-value; *p* = level of significance, two-tailed.

*Table S4.*

**Overview of strategies to reduce fear and discomfort in children undergoing an MRI examination**

| **Minimizing risks during research MRI** | **Minimizing burden during research MRI** |
| --- | --- |
| Asking for MRI compatibility via questionnaire:   - mechanical and electronical implants in the body, shard metal - previous surgeries - allergies - tattoos, permanent make-ups, piercings | Familiarizing the child to the MRI environment in advance:   - send comic to read at home illustrating the MRI procedure - practice at the hospital in a mock scanner - present video and audio recordings of the scanner and its surrounding - listen to other children talking about their MRI experience |
| Remove all metal objects:   - clothes with metallic objects (i.e. buttons, applications) - watch, piercings, ear rings, hair tie - content of all pockets (i.e. keys, coins, toys) | Reduce discomfort:   - use earplugs, headphones and/or cushions to reduce scanner noise - watch a relaxing video during the scan (i.e. nature documentaries, children’s movies) - listen to relaxing music |
| In participants of child-bearing age:   - exclude pregnancy via pregnancy test | Plan the scanning procedure as child-friendly as possible:   - allow for long preparation time - limit scanning time - allow breaks between sequence to talk to the child over intercom - let parents or study nurse stand next to the child during MRI - keep physical contact during MRI (hold hands or touch the leg) - honor the child’s courage and effort (i.e. self-made award, medal, certificate of MRI) |
| Accompanying parents standing next to their child:   - ensure that they are save for MRI (same criteria as for study participants) | Allow the child to have a feeling of control:   - stop the procedure immediately if the child present concerns (over button press) - allow the child to bring a stuffed animal as a guard - let the child talk to you after every sequence - let the child decide about the presence of accompanying persons |
|  | Use special interventions:   - presence of a hospital clown - animal assisted activities - virtual reality MR |
